# The autism and schizophrenia associated gene CYFIP1 is critical for the maintenance of dendritic complexity and the stabilization of mature spines

**DOI:** 10.1038/tp.2014.16

**Published:** 2014-03-25

**Authors:** M Pathania, E C Davenport, J Muir, D F Sheehan, G López-Doménech, J T Kittler

**Affiliations:** 1 Department of Neuroscience, Physiology and Pharmacology, University College London, London, UK

## Abstract

Copy number variation (CNV) at the 15q11.2 region has been identified as a significant risk locus for neurological and neuropsychiatric conditions such as schizophrenia (SCZ) and autism spectrum disorder (ASD). However, the individual roles for genes at this locus in nervous system development, function and connectivity remain poorly understood. Haploinsufficiency of one gene in this region, *Cyfip1*, may provide a model for 15q11.2 CNV-associated neuropsychiatric phenotypes. Here we show that altering CYFIP1 expression levels in neurons both *in vitro* and *in vivo* influences dendritic complexity, spine morphology, spine actin dynamics and synaptic α-amino-3-hydroxy-5-methyl-4-isoxazolepropionic acid (AMPA) receptor lateral diffusion. CYFIP1 is highly enriched at synapses and its overexpression *in vitro* leads to increased dendritic complexity. Neurons derived from *Cyfip1* heterozygous animals on the other hand, possess reduced dendritic complexity, increased mobile F-actin and enhanced GluA2-containing AMPA receptor mobility at synapses. Interestingly, *Cyfip1* overexpression or haploinsufficiency increased immature spine number, whereas activity-dependent changes in spine volume were occluded in *Cyfip1* haploinsufficient neurons. *In vivo*, *Cyfip1* heterozygous animals exhibited deficits in dendritic complexity as well as an altered ratio of immature-to-mature spines in hippocampal CA1 neurons. In summary, we provide evidence that dysregulation of CYFIP1 expression levels leads to pathological changes in CNS maturation and neuronal connectivity, both of which may contribute to the development of the neurological symptoms seen in ASD and SCZ.

## Introduction

Copy number variations of the 15q11.2 region of the human genome are implicated in the development of neurological and neuropsychiatric conditions such as autism spectrum disorder (ASD), epilepsy, intellectual disability (ID) and schizophrenia (SCZ).^1, 2, 3, 4, 5, 6, 7^ Copy number variation of one gene within 15q11.2, coding for the cytoplasmic FMRP-interacting protein 1 (CYFIP1), has been linked to both ASD and SCZ.^5,8,9^ Furthermore, genome-wide expression profiling of patients with duplication of 15q11–q13 has specifically demonstrated upregulated *Cyfip1* mRNA in those that suffer from ASD.^10^ Changes in CYFIP1 levels may thus lead to the neuropsychiatric and cognitive phenotypes associated with copy number variation at 15q11.2. However, the exact role that CYFIP1 has in normal nervous system function and development, and the consequences of its dysregulation, remain poorly understood.

Dendritic morphogenesis, synaptogenesis and continued activity-dependent remodelling of synaptic connections are critical for the development and maintenance of functional neuronal networks.^11, 12, 13, 14, 15^ A disruption in any of these processes produces network-wide deficits in neuronal connectivity and may help explain why changes in morphological complexity, dendritic spine number, shape and plasticity are associated with several neuropsychiatric and neurological disorders, including SCZ, ASD, ID and epilepsy.^16, 17, 18, 19^ Actin cytoskeletal dynamics have a key role in the establishment and maintenance of both dendritic arborizations and spines, and are also critical for the structural alterations in spine shape important for cellular forms of synaptic plasticity, such as long-term potentiation and long-term depression.^20, 21, 22, 23^ Moreover, alterations in the regulation of actin at dendritic spines, by neurodevelopmental disease-associated proteins such as DISC1, DTNBP1, SHANK3, PAK3, Oligophrenin and SRGAP2, have been shown to produce deficits in both synaptic plasticity and the formation and consolidation of long-term memories.^23, 24, 25, 26, 27, 28, 29^

One critical modulator of cellular actin dynamics is the ubiquitous heteropentameric WAVE regulatory complex consisting of the proteins WAVE, Abi, Nap1, HSPC300 and CYFIP1 (also known as SRA-1) or CYFIP2.^30^ The main role of CYFIP1 in this complex is to maintain the WAVE regulatory complex in an inhibited state until the small GTPase Rac1, once activated, binds CYFIP1. Rac1 binding results in dissociation of CYFIP1 from the WAVE regulatory complex, allowing WAVE to activate the actin nucleator Arp2/3 and subsequently enabling *de novo* F-actin assembly.^30,31^ In this way, CYFIP1 functions as a key, conserved regulator of actin nucleation in cells.^30^ Furthermore, CYFIP1 couples actin dynamics to other cellular processes, such as carrier vesicle biogenesis in the trans-Golgi network, and, via interactions with the FRMP (Fragile X mental retardation protein), regulates synaptic mRNA translation in an activity-dependent manner.^32,33^ However, still very little is known about CYFIP1's role in establishing or maintaining neuronal connectivity, specifically its involvement in the regulation of dendritic morphology, synaptic structural plasticity and neurotransmitter receptor mobility. Furthermore, how both deletions and duplications affecting *Cyfip1* expression produce similar neuropsychiatric phenotypes has also not yet been addressed.^34,35^

Here we show that both CYFIP1 and CYFIP2 are highly enriched at excitatory synapses in dendritic spines. In dissociated neurons, overexpression of CYFIP1 or CYFIP2 leads to increased dendritic complexity and altered spine morphology whereas conversely, reducing CYFIP1 levels causes decreased dendritic complexity and an increase in immature dendritic spines. CYFIP1 deficiency results in increased F-actin assembly within dendritic spines, occludes N-methyl-D-aspartate (NMDA) receptor-induced spine shrinkage and leads to alterations in the surface dynamics and synaptic mobility of α-amino-3-hydroxy-5-methyl-4-isoxazolepropionic acid (AMPA) receptors. *In vivo*, *Cyfip1* deletion in the mouse leads to delayed embryonic development and early embryonic death whereas *Cyfip1* haploinsufficiency causes reduced dendritic complexity and an increase in immature spines in the hippocampus. Our results support a critical role for CYFIP1 in development, dendritic morphogenesis and synaptic connectivity and provide further support for the idea that dysregulated CYFIP1 mediates the molecular aetiology of 15q11.2 Copy number variation-associated neuropsychiatric conditions such as ASD, SCZ and epilepsy.

## Materials and Methods

### Cell culture and transfection

Hippocampal cultures were obtained from E16 mouse embryos (produced via *Cyfip1*^+/+^x*Cyfip1*^+/^^−^ crosses) or E18 rat embryos as previously described and transfected using Lipofectamine-2000 (Life Technologies, Paisley, UK).^36^

### Microscopy, quantitative analysis of neuronal and dendritic spine morphology, and fluorescence recovery after photobleaching (FRAP)

Confocal images were acquired on a Zeiss LSM700 upright confocal microscope using a 63X oil objective (NA: 1.4) unless otherwise stated (Carl Zeiss, Welwyn Garden City, UK). Image stacks for dendritic morphological and spine analysis had voxel dimensions of 0.39 μm × 0.39 μm × 0.54 μm and 0.19 μm × 0.19 μm × 0.57 μm, respectively. Neuronal arbours were reconstructed and subjected to Sholl analysis, using either Imaris (Bitplane AG, Zurich, Switzerland) or NeuronStudio and ImageJ (NIH, Bethesda, MD, USA).^37^ Spines were manually identified on 100–200 μm long secondary dendrites and analyzed for morphological classification or volume measurements in Imaris software (Bitplane). For spine classification custom parameters were used and spines were classified into stubby, mushroom, long and thin, and filopodia categories using a ratio of spine head and neck diameters to spine length. Classification was entirely automated until the final step where errors in classification were removed.

Single-plane confocal images were acquired for localization experiments *in vitro*. To quantify CYFIP1 and CYFIP2 enrichment at synaptic sites, the fluorescence intensity of CYFIP1 or CYFIP2 was measured as the average intensity within the labelled synaptic puncta and normalized to the average intensity of the total process including synaptic puncta using Metamorph software (Molecular Devices, Sunnyvale, CA, USA).

Dendritic morphology and spines in P55–60 mice were analysed using the FD Rapid Golgi Stain kit (FD NeuroTechnologies, Baltimore, MD, USA) and Neurolucida (MBF Bioscience, Williston, VT, USA). Golgi-impregnated brains were sliced at 150 μm using a vibratome (Leica Microsystems, Heerbrugg, Switzerland). Well-isolated hippocampal CA1 neurons were imaged at 60X using the Neurolucida software system and an upright light microscope with a motorized stage (MBF Bioscience). The entire extent (apical and basal) of the dendritic tree was traced and reconstructed. For spine analysis, two apical oblique and two basal dendrites were quantified per cell. Three males were analysed for each condition with a minimum of three cells traced per animal. When using animals from separate litters, each genotype was equally represented from each litter.

Chemical long-term depression and fluorescence recovery after photobleaching (FRAP) on actin in dendritic spines was performed as described previously.^38,39^ FRAP movies were analysed in ImageJ (NIH) using a custom-built plugin, curves were fitted using *Mathematica* (Wolfram Research, Champaign, IL, USA). For further details see Supplementary Methods.

### Quantum dot (QD) imaging

A total of 12–16DIV hippocampal cells transfected with GluA2^SEP^ were live imaged under perfusion in ACSF at 37 °C and quantum dot (QD) movies were recorded at 8.5 Hz. Labelling of GluA2^SEP^ with QDs was performed at RT using a mouse anti-GFP antibody (10 μg ml^−1^, Neuromab, N38) and an anti-mouse 605-nm QD (0.5 nM, Life Technologies). Analysis of QD receptor dynamics was performed as described (see Supplementary Methods).^40^

## Results

### CYFIP1 and CYFIP2 are enriched at excitatory synapses

**Figure 1:**
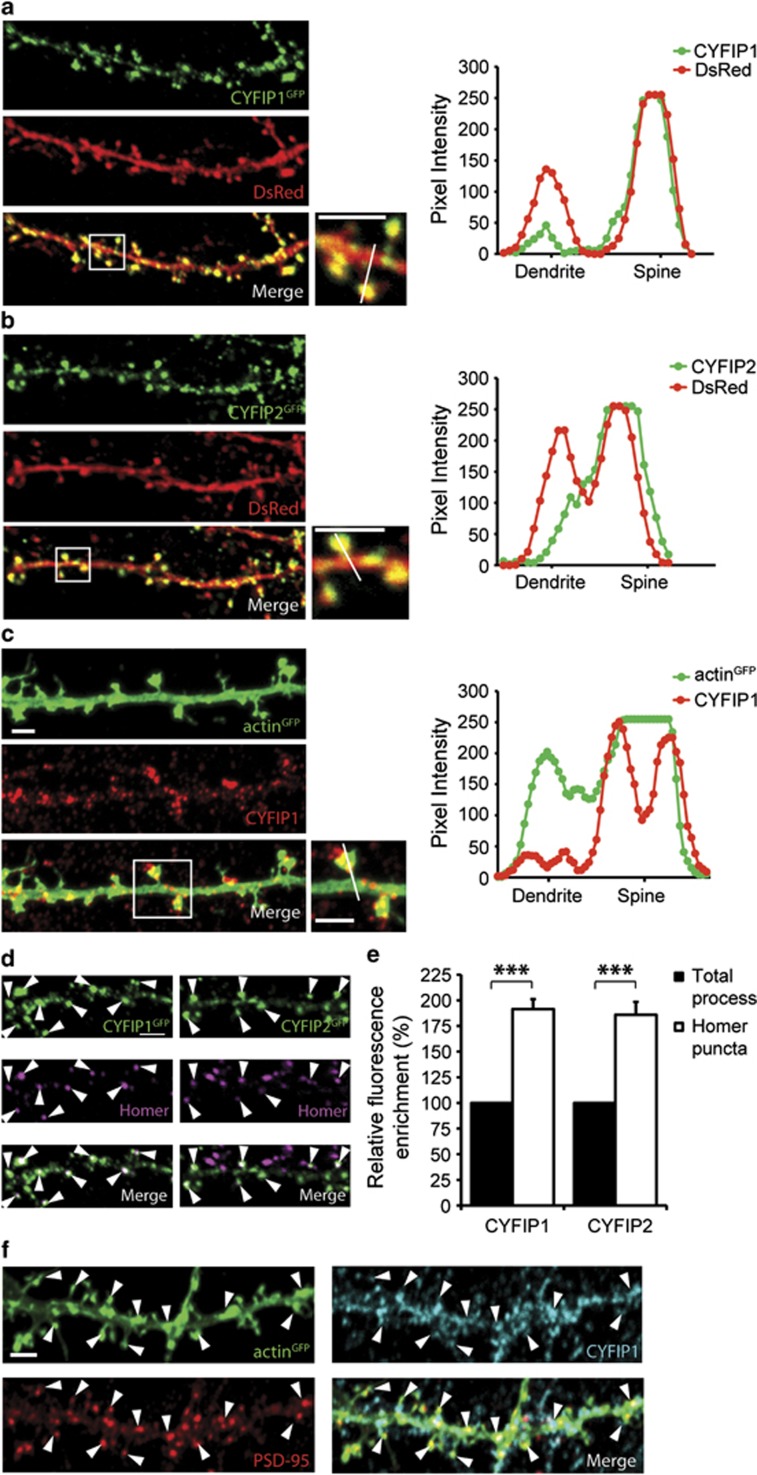
CYFIP1 and CYFIP2 are localized at dendritic spines and enriched at excitatory synapses. In 17–20DIV rat hippocampal neurons, CYFIP1^GFP^ (**a**), CYFIP2^GFP^ (**b**) or endogenous CYFIP1 (**c**) localize to dendritic spines. DsRed (**a**, **b**) or actin^GFP^ (**c**) were used to label processes. A line-scan through the dendritic shaft and spine head (right graphs) shows the fluorescence intensity of the green and red channels depicting the enrichment of CYFIP1^GFP^, CYFIP2^GFP^ or endogenous CYFIP1 in the spine compared with the dendrite. CYFIP1^GFP^ and CYFIP2^GFP^ clusters co-localize with the excitatory synaptic marker Homer (**d**) (arrowheads). Quantification shows that there is an enrichment of CYFIP1^GFP^ and CYFIP2^GFP^ at excitatory synapses compared with the total process (excitatory: CYFIP1 *n*=61, CYFIP2 *n*=54, ***P<0.001) (**e**). Endogenous CYFIP1 also co-localizes with the excitatory synaptic marker PSD-95 (arrowheads) in actin^GFP^-expressing cells (**f**). Scale bars, 2 μm.

To explore the localization of CYFIP1 and CYFIP2 in neurons CYFIP1^GFP^ or CYFIP2^GFP^ (Supplementary Figure 1) were co-expressed in mature hippocampal neurons with DsRed as a marker of neuronal morphology and analysed using confocal microscopy. Compared with the localization of DsRed, which uniformly filled both dendritic processes and spines, CYFIP1^GFP^ and CYFIP2^GFP^ exhibited a nonuniform distribution appearing to be selectively targeted to punctate clusters in spines and along dendrites (Figures 1a and b). A similar enrichment in spines was observed for endogenous CYFIP1 using a CYFIP1-specific antibody and actin^GFP^ to label neuronal morphology (Figure 1c; Supplementary Figure 1). In addition, CYFIP1 is detected within the post synaptic density fraction by western blotting (Supplementary Figure 4). Co-expression experiments with CYFIP1^GFP^ and CYFIP2^mCherry^ revealed that the majority of CYFIP1^GFP^ co-localized with CYFIP2^mCherry^ and overlapping puncta were found both in dendritic processes and spines (Supplementary Figure 2A).

Using immunofluorescence and confocal microscopy, both CYFIP1^GFP^ and CYFIP2^GFP^ levels were found to be ~90% enriched at Homer and VGLUT-positive excitatory synaptic sites compared with total dendritic processes (CYFIP1, 91.4±9.8% increase, *P*<0.001; CYFIP2, 85.9±12.6% increase, *P*<0.001; Figures 1d and e,Supplementary Figure 2B). In addition, endogenous CYFIP1 was also highly enriched at excitatory synapses and co-localized with the excitatory marker PSD-95 (Figure 1f). Thus endogenous CYFIP1, along with CYFIP1^GFP^ and CYFIP2^GFP^, are found at excitatory synapses and exhibit high levels of expression at sites of F-actin accumulation, like dendritic spines.

### Overexpression of CYFIP1 and CYFIP2 enhances dendritic complexity and alters dendritic spine morphology

**Figure 2:**
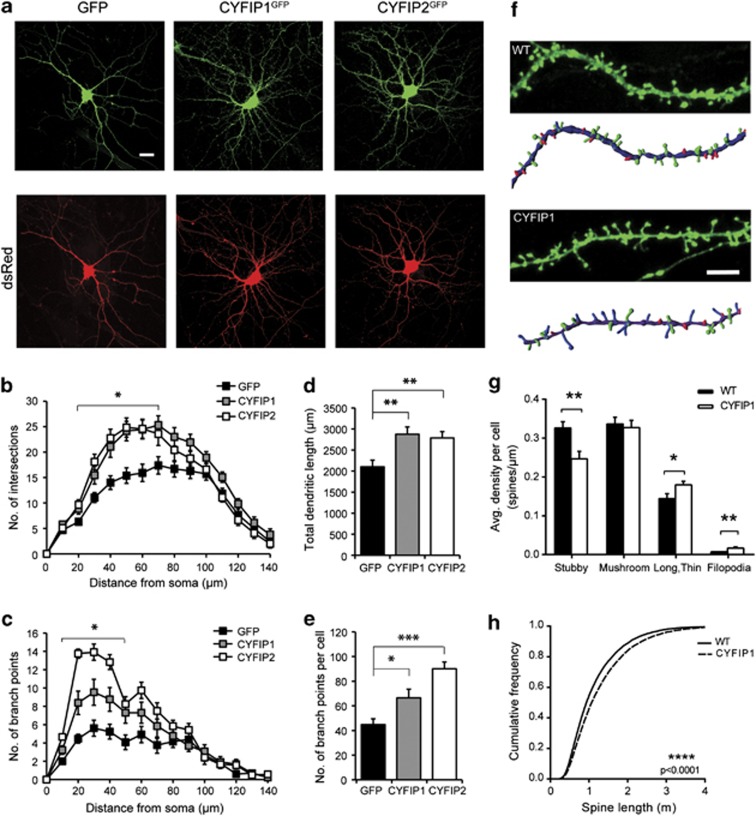
CYFIP1 and CYFIP2 promote increased dendritic complexity and altered dendritic spine structure. (**a**) Overexpression of CYFIP1^GFP^ or CYFIP2^GFP^ for 4 days results in increased dendritic complexity compared with expression of control GFP in 14DIV rat hippocampal neurons co-transfected with DsRed. Scale bar, 20 μm. Quantification by Sholl analysis shows that number of intersections (**b**) and number of branch points (**c**) are significantly increased with distance from the soma in CYFIP1- or CYFIP2-overexpressing neurons compared with GFP control (data points represent an average of 12–13 cells, *P<0.05). Expression of CYFIP1 or CYFIP2 increases total dendritic length (**d**) (CYFIP1, *n*=13, **P<0.01, CYFIP2, *n*=12, **P<0.01, compared with control *n*=13) and number of branch points per cell (**e**) (CYFIP1, *n*=13, *P<0.05, CYFIP2, *n*=12, ***P<0.001, compared with control *n*=13). Spine morphology was analysed at 21DIV in CYFIP1^mCherry^+actin^GFP^-expressing mouse hippocampal neurons and compared with DsRed+actin^GFP^-expressing cells (**f**) (upper panel: representative image; lower panel: 3D reconstruction). Scale bar, 5 μm. Colour key for spine type in 3-dimensional reconstruction: green=mushroom, red=stubby, blue=long and thin, pink=filipodia. CYFIP1^mCherry^ overexpression resulted in decreased stubby spines and increased long, thin spines and filopodia (**g**) (*n*=16–18 cells per condition, *P<0.05, **P<0.01). Spine length is increased across the entire population regardless of subtype classification in CYFIP1-overexpressing neurons compared with control neurons (**h**) (*n*=15 000–19 000 spines per condition, ****P<0.0001).

Microduplication of the CYFIP1-encoding region at 15q11.2 is associated with increased neuropsychiatric disease burden, and increased expression of CYFIP1 has specifically been linked to ASD.^1,3,10^ Overexpression of CYFIP1^GFP^ or CYFIP2^GFP^ resulted in a significant increase in dendritic complexity as a function of distance from the soma compared with control neurons expressing GFP, measured using either number of intersections or number of branch points as indicators of morphological complexity (Figures 2a–c). In addition, total dendritic length and the total number of branch points per cell were also increased in the CYFIP1 and CYFIP2 overexpressors (dendritic length: CYFIP1, 37% increase, 2876±174 μm, *P*<0.01; CYFIP2, 33% increase, 2787±152 μm, *P*<0.01; GFP, 2099±163 μm; branch points: CYFIP1, 48% increase, 66.5±7.0, *P*<0.05; CYFIP2, 101% increase, 90.1±5.6, *P*<0.001; GFP 44.8±7.7; Figures 2d and e).

To examine whether changes in dendritic complexity were accompanied by changes in excitatory spine number or shape, we measured spines in CYFIP1^mCherry^+actin^GFP^-expressing neurons at 21DIV. Compared with DsRed+actin^GFP^ transfected neurons, CYFIP1^mCherry^+actin^GFP^-expressing cells displayed no significant difference in spine density (data not shown). However, CYFIP1 overexpressors had significantly fewer stubby spines (30% decrease in stubby spines, DsRed+actin^GFP^: 0.32±0.01 spines μm^−1^, CYFIP1^mCherry^+actin^GFP^: 0.24±0.01 spines μm^−1^, *P*<0.01) and increased numbers of long, thin spines and filopodia (24% increase in long, thin spines, DsRed+actin^GFP^: 0.14±0.01 spines μm^−1^, CYFIP1^mCherry^+actin^GFP^: 0.17±0.01 spines μm^−1^, *P*<0.05; 145% increase in filopodia, DsRed+actin^GFP^: 0.006±0.001 spines μm^−1^, CYFIP1^mCherry^+actin^GFP^: 0.016±0.003 spines μm^−1^, *P*<0.01; Figures 2f and g). Furthermore, spines were significantly longer in CYFIP1 overexpressors regardless of subtype classification (DsRed+actin^GFP^: 1.13±0.005 μm, CYFIP1^mCherry^+actin^GFP^: 1.29±0.005 μm, *P*<0.0001, Figure 2h). CYFIP1 overexpression also significantly decreased spine diameter, whereas spine volume showed an overall increase due to the dominating effect of the increase in spine length (Supplementary Figures 6C and D). These results demonstrate that increasing CYFIP1 expression levels above baseline enhances dendritic complexity and alters the ratio of mature versus immature spines in neurons, thereby changing network connectivity.

### CYFIP1 is essential for embryonic development and its loss leads to marked developmental delay

To study the effects of decreased *Cyfip1* levels *in vivo*, we characterized a *Cyfip1* KO mouse^41^ and found that deletion was embryonically lethal. However, fertilized KO oocytes from *Cyfip1*^+/^^−^ × *Cyfip1*^+/^^−^ crosses did persist up to the blastocyst stage *in vitro* (not shown), and in pregnant dams, they were detectable until embryonic day 8.5 (E8.5) postcoitum (Supplementary Table 1). At this stage, *Cyfip1* KO embryos were significantly reduced in size and displayed delayed development compared with their heterozygous (*Cyfip1*^+/^^−^) or wild-type (WT) counterparts (Supplementary Figures 3A–C). Further examination revealed complete developmental failure resulting in reduced body elongation and patterning. *Cyfip1*^*+/*^^−^ heterozygous animals on the other hand, were viable until adulthood, were fertile and did not show any gross morphological abnormalities in brain structure when compared with WT (Supplementary Figure 3H). These data suggest that adequate levels of CYFIP1 are indispensable for proper development through early stages of embryogenesis.

### *Cyfip1* haploinsufficiency results in decreased dendritic complexity and increased immature spine number

**Figure 3:**
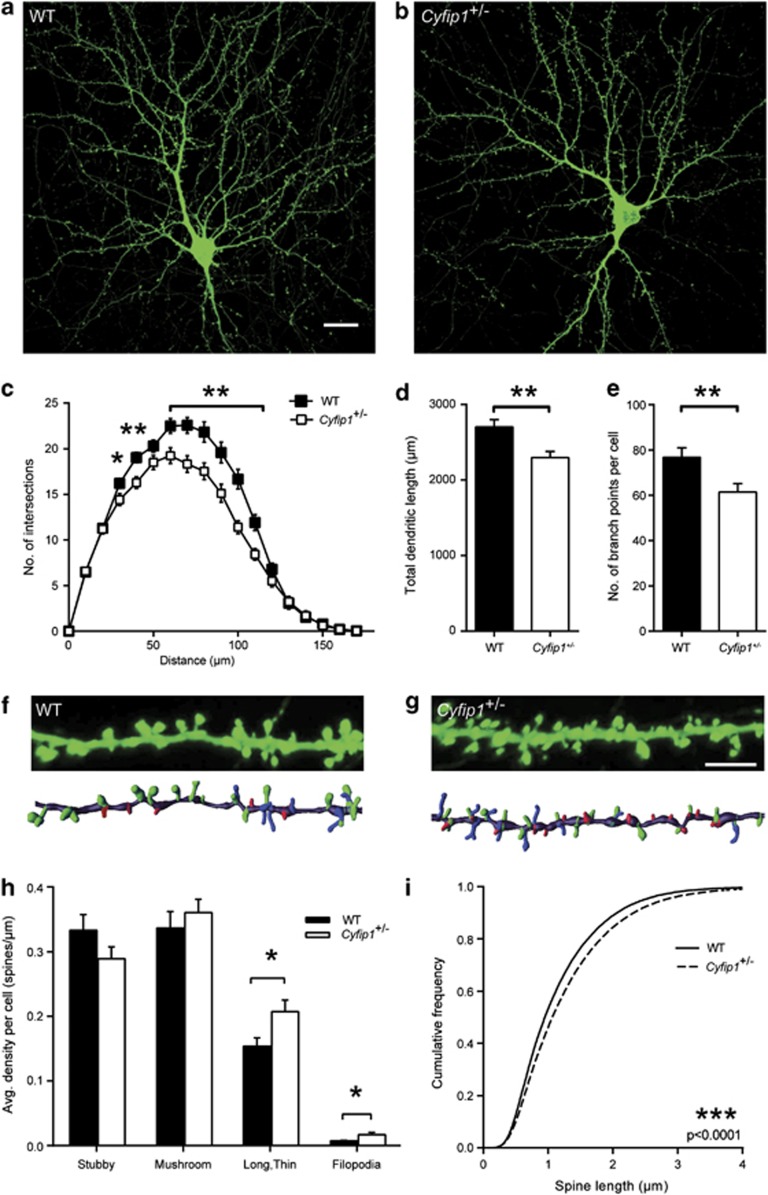
*Cyfip1* haploinsufficient neurons display decreased dendritic complexity and increased immature spines. *Cyfip1*^+/^^−^ hippocampal neurons had reduced dendritic complexity compared with WT controls at 14DIV (**a**, **b**). Scale bar, 20 μm. Dendritic morphology was examined using Sholl analysis. *Cyfip1*^+/^^−^ neurons show significantly less complex morphology as a function of distance from the soma when compared with WT neurons (*n*=18 cells per condition, *P<0.05, **P<0.01) (**c**). Total dendritic length (**d**) and total number of branch points (**e**) (*n*=18 cells per condition, **P<0.01) are also reduced in *Cyfip1*^+/^^−^ neurons compared with WT. Spine morphology was analysed at 21DIV in *Cyfip1*^+/^^−^ neurons and compared with WT cells (**f**, **g**) (upper panel: representative image; lower panel: 3-dimensional reconstruction). Scale bar, 5 μm. Colour key for spine type in 3-dimensional reconstruction: green=mushroom, red=stubby, blue=long and thin, pink=filipodia. *Cyfip1*^+/^^−^ neurons possessed increased long, thin spines and filopodia (**h**) (*n*=27–31 cells per condition, *P<0.05). Spine length is increased across the entire population regardless of subtype classification in *Cyfip1*^+/^^−^ neurons compared with control neurons (**i**) (*n*=~39 000 spines per condition, ***P<0.001).

Microdeletion at 15q11.2 renders people haploinsufficient for *Cyfip1* and is associated with ASD and SCZ.^4,6,7^ We therefore investigated the effects of decreased CYFIP1 levels by measuring dendritic and spine morphologies in *Cyfip1*^+/^^−^ hippocampal neurons (Supplementary Figures 3F and G). Sholl analysis of 14DIV, actin^GFP^-labelled neurons revealed that reduced CYFIP1 levels had the opposite effect on dendritic complexity to that of CYFIP1 overexpression, leading to a marked decrease in dendritic complexity between 30 and 110 μm away from the soma (Figures 3a–c). Furthermore, both total dendritic length and the total number of branch points were reduced in *Cyfip1*^+/^^−^ neurons compared with control (15% decrease in total dendritic length in *Cyfip1*^+/^^−^ neurons, WT: 2704±96.8 μm, *Cyfip1*^+/^^−^: 2298±80.2 μm, *P*<0.01; 20% decrease in number of branch points in *Cyfip1*^+/^^−^ neurons, WT: 76.7±4.1, *Cyfip1*^+/^^−^: 61.4±3.6, *P*<0.01; Figures 3d and e). Spine density was unchanged in *Cyfip1*^+/^^−^ neurons compared with WT controls; however, when spines were further classified into the different morphological subtypes, long and thin spines and filopodia were significantly increased (30% increase in long, thin spines, WT: 0.15±0.01 spines μm^−1^, *Cyfip1*^+/^^−^: 0.20±0.01 spines μm^−1^, *P*<0.05; 125% increase in filopodia, WT: 0.007±0.001 spines μm^−1^, *Cyfip1*^+/^^−^: 0.016±0.003 spines μm^−1^, *P*<0.05, respectively; Figures 3f–h). In addition, spines were significantly longer in the *Cyfip1*^+/^^−^ neurons irrespective of subtype classification (WT: 1.14±0.003 μm, *Cyfip1*^+/^^−^: 1.27±0.003 μm, *P*<0.0001, Figure 3i). However, no change in spine diameter or volume was observed in the *Cyfip1* haploinsufficient neurons (Supplementary Figures 6A and B). These experiments demonstrate that *Cyfip1* haploinsufficiency results in decreased dendritic complexity and produces alterations in spine morphology.

### CYFIP1 regulates actin assembly at dendritic spines

**Figure 4:**
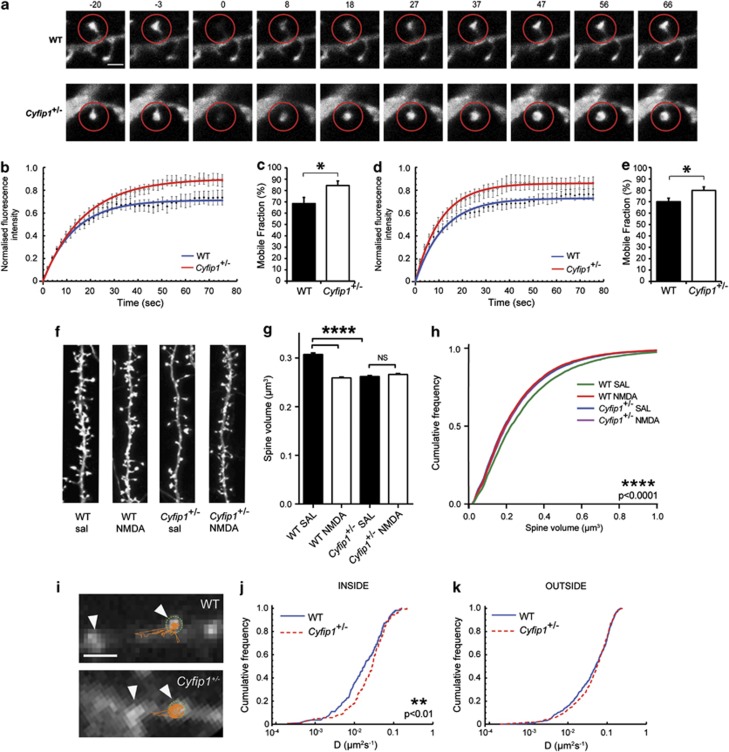
*Cyfip1* deficiency affects F-actin dynamics in spines, occludes structural responses to cLTD and increases surface AMPA receptor mobility. *Cyfip1*^+/^^−^ and WT hippocampal neurons were transfected with actin^GFP^ or Lifeact^GFP^ at 17–20DIV and subjected to FRAP live imaging 2 days later. Spines were imaged for 100 s and bleaching occurred after the first 20 s. (**a**) Representative images over time in seconds of actin^GFP^ fluorescence recovery in WT and *Cyfip1*^+/^^−^ spines. The red circles highlight the bleached spine. Scale bar, 2 μm. Quantification of GFP fluorescence intensity within the spine head region of WT compared with *Cyfip1*^+/^^−^ neurons transfected with actin^GFP^ (**b**) or Lifeact^GFP^ (**d**) shows *Cyfip1*^+/^^−^ spines recover to a greater extent than WT (data points represent an average of 25–33 movies, *P<0.05). Data are fitted with single exponentials (coloured lines). The mobile fraction, quantified as the final amount of recovered fluorescence presented as a percentage of the total bleached fluorescence, is significantly increased in *Cyfip1*^+/^^−^ neurons for both actin^GFP^ (**c**) (*n*=29–33, *P<0.05) and Lifeact^GFP^ (**e**) (*n*=24–29, *P<0.05). 21DIV WT and *Cyfip1*^+/^^−^ hippocampal neurons were transfected with actin^GFP^ and treated with either 20 μM NMDA+20 μM glycine (NMDA) or saline (SAL). (**f**) Representative images of spines following each treatment. Scale bar, 5 μm. Spine volume is significantly decreased in WT neurons following NMDA treatment compared with saline (*n*=8000–13 000 spines per condition, ****P<0.0001). However, *Cyfip1*^+/^^−^ spines have basally low volumes and fail to remodel in response to NMDA (*n*=12 000–14 000 spines per condition, ****P<0.0001) (**g**). Spine volume is significantly decreased in WT NMDA-treated neurons compared with saline treatment whereas *Cyfip1*^+/^^−^ saline- and NMDA-treated neurons show no leftward shift (*n*=8000–14 000 spines, ****P<0.0001, one-way analysis of variance (ANOVA)) (**h**). GluA2^SEP^-containing receptors were labelled with quantum dots (QDs) and live imaged. (**i**) Representative QD trajectories are shown for WT and *Cyfip1*^*+/*^^−^ neurons, arrowheads indicate GluA2^SEP^-containing synapses and green dotted line indicates synapse area. Scale bar, 2 μm. Quantification of receptor diffusion inside (**j**) and outside (**k**) synaptic clusters revealed that GluA2^SEP^-containing receptors are more mobile in synaptic clusters of *Cyfip1*^+/^^−^ neurons compared with WT (*n*=140–160 QD tracks, **P<0.01). cLTD, chemical long-term depression.

CYFIP1 negatively regulates local actin filament assembly, suggesting that actin dynamics may be altered in *Cyfip1*^+/^^−^ dendritic spines. FRAP of actin^GFP^ in spines of *Cyfip1*^*+/*^^−^ neurons revealed a significant difference in amount of fluorescence recovery compared with WT controls. Fluorescence intensity within the spine head of WT neurons returned to only 64.1±5.2% of the pre-bleached fluorescence by 40 s compared with 80.0±4.1% in the *Cyfip1*^+/^^−^ neurons (*P*<0.05; Figures 4a and b). Moreover, the fluorescence recovery of *Cyfip1*^+/^^−^ neurons plateaued at a greater intensity than WT neurons, which was confirmed by a significant increase in the total mobile fraction (i.e. the proportion of final recovered fluorescence compared with the total bleached fluorescence, 16% increase, WT: 68.4±5.46%, *Cyfip1*^+/^^−^: 84.4±3.97%, *P*<0.05; Figure 4c). However, there was no change in the recovery rate constant between *Cyfip1*^*+/*^^−^ and WT neurons. Similar results were also obtained using Lifeact^GFP^, an F-actin-specific fluorescent probe suitable for FRAP experiments.^42^ Photobleaching spines expressing Lifeact^GFP^ produced a significantly greater amount of recovered fluorescence in *Cyfip1*^+/^^−^ neurons compared with WT again by 40 s (WT: 67.9±4.8%, *Cyfip1*^+/^^−^: 85.5±7.0%, *P*<0.05; Figure 4d) resulting in a significant increase in the total mobile fraction (9.8% increase, WT: 70.1±3.03%, *Cyfip1*^+/^^−^: 79.9±3.12%, *P*<0.05; Figure 4e).^42,43^ Taken together, the increased recovery of actin^GFP^ and Lifeact^GFP^ suggests an increase in F-actin assembly in the *Cyfip1*^+/^^−^ spines.

### *Cyfip1* haploinsufficiency occludes NMDA receptor-dependent structural plasticity

As *Cyfip1* haploinsufficiency changes actin availability in spines and alters the ratio of mature versus immature spines at steady-state, we hypothesised that stimulus-dependent structural plasticity would also be altered in *Cyfip1*^+/^^−^ spines. To study the role that CYFIP1 has in spines during structural plasticity, we utilized an established chemical long-term depression protocol.^38^ Brief bath application of 20 μM NMDA plus 20 μM glycine induces a 15–20% spine shrinkage within a timeframe of 40 min, which is measured as a decrease in spine volume without a concomitant change in either spine diameter or length (Figures 4f and g; Supplementary Figures 6E and F). Using this stimulus, we were able to induce spine shrinkage in WT neurons in response to NMDA treatment (Figures 4f and g). Cumulative frequency curves of spine volume show a leftward shift following NMDA application in WT neurons, indicating a reduction of spine volume across the population (Figure 4h). *Cyfip1*^+/^^−^ spines showed no change in spine volume following NMDA application and spine volume remained unchanged from its basally low levels (16% decrease in WT spine volume following NMDA treatment, no change in *Cyfip1*^+/^^−^ spine volume following NMDA treatment, WT saline: 0.30±0.003 μm^3^, WT NMDA: 0.25±0.001 μm^3^, *Cyfip1*^+/^^−^ saline: 0.26±0.001 μm^3^, *Cyfip1*^+/^^−^ NMDA: 0.26±0.002 μm^3^, *P*<0.0001; Figures 4f–h). These data reveal a role for CYFIP1 in mediating structural reorganization during synaptic plasticity, possibly through dynamic regulation of the local actin cytoskeleton in spines.

### Altered surface AMPA receptor mobility due to *Cyfip1* haploinsufficiency

AMPA receptors mediate fast excitatory synaptic transmission within the CNS.^44,45^ Actin dynamics in spines have a major role in coordinating the delivery and removal of AMPA receptors and the strength of synapses.^45^ To address whether CYFIP1-mediated alterations in spine morphology and actin dynamics also impinge on the stability or mobility of AMPA receptors within synapses, we investigated whether surface GluA2-containing receptor dynamics were different from control in *Cyfip1*^+/^^−^ neurons. Initial observations suggested that total protein levels of the AMPA receptor subunit GluA2 are unchanged in WT and *Cyfip1*^+/^^−^ neurons (Supplementary Figures 5A and B). Therefore, we expressed pH-sensitive superecliptic pHluorin (SEP)-tagged GluA2 in neurons to visualize GluA2-containing synaptic clusters in dendrites. Single GluA2^SEP^-containing receptors were labelled with 605 nm QDs to follow the diffusion of individual receptors within synaptic clusters.^40^ QD-labelled GluA2^SEP^ receptors were less mobile when inside synaptic clusters (*D*_in_=0.023 μm^2^ s^−1^, *D*_out_=0.057 μm^2^ s^−1^ where *D* is the median diffusion coefficient, *P*<0.0001, Supplementary Figures 5C and D), indicating confined motion at synapses, as previously shown.^46^ In *Cyfip1*^+/^^−^ neurons, however, GluA2^SEP^-containing receptors exhibited an increased synaptic mobility within clusters when compared with WT neurons, (WT: *D*_in_=0.023 μm^2^ s^−1^, *Cyfip1*^+/^^−^: *D*_in_=0.034 μm^2^ s^−1^, *P*<0.01; Figures 4i and j) as well as a reduced confinement at synapses (Supplementary Figure 5E). GluA2^SEP^ receptor mobility outside synapses remained unchanged between WT and *Cyfip1*^+/^^−^ neurons (WT: *D*_out_=0.057 μm^2^ s^−1^, *Cyfip1*^+/^^−^: *D*_out_=0.062 μm^2^ s^−1^, Figure 4k). These observations suggest that the restricted mobility of GluA2 receptors at synapses is significantly diminished in *Cyfip1*^+/^^−^ spines.

### CYFIP1 regulates neuronal development and connectivity *in vivo*

**Figure 5:**
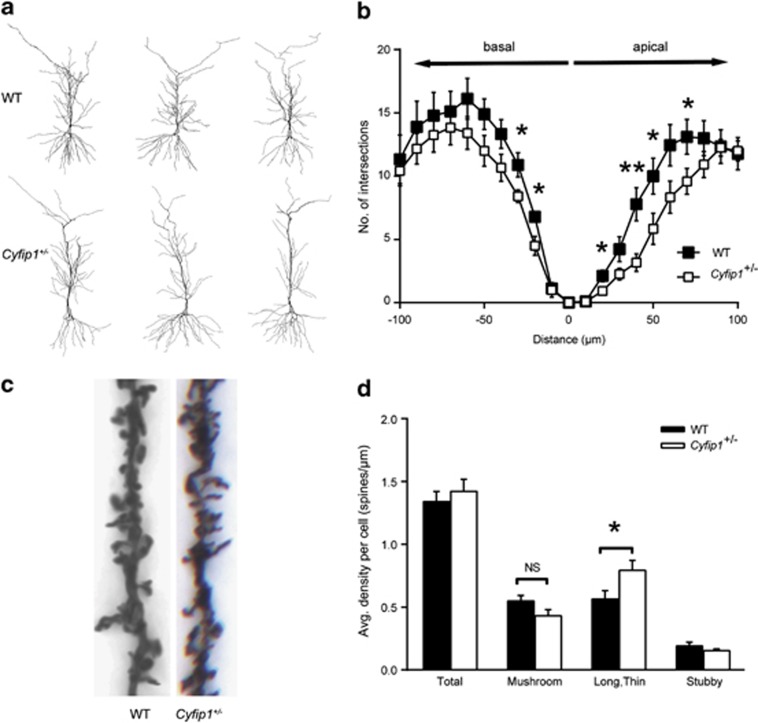
*Cyfip1* haploinsufficiency *in vivo* decreases dendritic complexity and increases the number of immature dendritic spines. Golgi-stained CA1 neurons from *Cyfip1*^+/^^−^ and WT littermate controls (P55–60) were traced to analyse dendritic morphology and spines. Example traces of *Cyfip1*^+/^^−^ and WT neurons (**a**). Sholl analysis indicates that *Cyfip1*^+/^^−^ neurons are less complex within 100 μm from the soma, in both apical and basal compartments, compared with WT control neurons (*n*=9–12 cells per condition, *P<0.05, **P<0.01) (**b**). Analysis of dendritic spine density reveals no change in total spine density but a significant increase in immature long, thin spines in *Cyfip1*^+/^^−^ neurons compared with WT control (*n*=12 cells per condition, *P<0.05) (**c**, **d**).

To confirm that *Cyfip1* haploinsufficiency produces similar effects on dendritic spines in the intact animal, we investigated dendritic complexity and spine morphology in the hippocampi of WT versus *Cyfip1*^+/^^−^ mice using Golgi staining. *Cyfip1* haploinsufficiency in postnatal day 55–60 (P55–60) animals was confirmed by western blotting (46.8% CYFIP1 protein decrease, WT control 100±15.4%, *Cyfip1*^+/^^−^ 53.2±7.8%, *P*<0.05; Supplementary Figures 3D and E). Compared with WT male littermate controls, CA1 neurons in *Cyfip1*^+/^^−^ animals possessed a significantly decreased dendritic complexity within 100 μm from the soma (Figures 5a and b). Furthermore, spine density was unchanged, but long and thin spines were increased in the apical oblique dendrites in *Cyfip1*^+/^^−^ animals (40% increase, WT: 0.56±0.066 spines μm^−1^, *Cyfip1*^+/^^−^: 0.79±0.078 spines μm^−1^, *P*<0.05; Figures 5c and d). These results indicate that genetic dysregulation of *Cyfip1* produces penetrant effects on pyramidal neuron dendritic complexity and spine morphology *in vivo*.

## Discussion

Altered levels of CYFIP1 are associated with several neurological disorders including SCZ and ASD.^4,5,10^ However, it remains unclear what role CYFIP1 has in the establishment or maintenance of neuronal connectivity in the mammalian CNS, and how changes in its expression contribute to neurological dysfunction. Here we report that CYFIP1 regulates the maturation of neuronal dendrites and dendritic spines, controls the availability of F-actin within spines and impacts both spine structural plasticity and mobility of AMPA receptors at synapses. Furthermore, modelling SCZ- and ASD-associated *Cyfip1* haploinsufficiency in mice produces deficits in dendritic complexity and spine morphology in hippocampal pyramidal neurons *in vivo*.

The activity of Rho GTPases (including RhoA, Rac1 and Cdc42) is associated with increased dendritic branch dynamics and extension.^20,21,47,48^ Activation of Rac1 releases the inhibitory action of CYFIP1 on WAVE, potentiating Arp2/3-mediated actin polymerization suggesting that changes in CYFIP1 levels could alter signalling events downstream of Rac1.^31^ Here we demonstrate that CYFIP1 expression levels are important for dendritic arborization and neuronal morphological complexity. Overexpression of CYFIP1 in hippocampal neurons *in vitro* leads to an increase in dendritic length and complexity whereas conversely, neurons from *Cyfip1*^+/^^−^ animals have smaller, less complex dendrites both *in vitro* and *in vivo*. Interestingly, in addition to the effects on dendritic morphogenesis that we report here, both WAVE1 and CYFIP1 have previously been implicated in axonal outgrowth.^49,50^

A strict spatiotemporal balance between actin polymerization and turnover, mediated by Rho GTPases, is also important for spine formation, maturation and stability.^20,21^ We report that CYFIP1 is highly enriched in dendritic spines where it co-localizes with actin. Thus, CYFIP1 is critically placed, as a key Rac1 effector, to locally regulate WAVE in spines, and variations in its expression levels are likely to impact on Arp2/3-dependent spine actin dynamics downstream of Rac1. In agreement with this, we find that downregulating CYFIP1 expression levels *in vitro* or *in vivo* drives an increase in the ratio of immature-to-mature spines suggesting that CYFIP1 is important for actin-dependent regulation of spine maturation. Furthermore, we show that dendritic spines from *Cyfip1*^+/^^−^ haploinsufficient neurons display enhanced F-actin availability suggesting that spatiotemporal control over actin polymerization is altered at *Cyfip1*^+/^^−^ postsynaptic sites. A role for CYFIP1 in the regulation of Rac1-dependent F-actin assembly in *Drosophila* NMJ presynaptic terminals was also recently demonstrated, where it was shown to be important for synaptogenesis and presynaptic quantal content.^51,52^ In addition to previous functions described for CYFIP1 in the regulation of mRNA translation in dendrites and mGluR-long-term depression, our results suggest that CYFIP1 also has a vital role in the local regulation of spine actin dynamics via WAVE and Arp2/3.^32,35^ This is in agreement with a recent study showing that CYFIP1 translocates between the FMRP-containing protein synthesis machinery and the actin-regulatory complex, and that disrupting CYFIP1's interaction with members of either complex negatively impacted spine maturation in neurons.^34^

The surface mobility of neurotransmitter receptors is dynamically regulated to control synaptic activity and is emerging as another key component underlying plasticity at nerve terminals.^40,53,54^ The trapping of mobile AMPA receptors within the postsynaptic domain is thought to be critical for maintaining and strengthening synaptic communication, and a pathological impairment in this process could have significant implications for neurotransmission.^55^ The enrichment of CYFIP1 at excitatory synapses implies that CYFIP1 can locally regulate actin cytoskeletal dynamics in the postsynaptic domain. This fine-tuning of actin turnover has previously been demonstrated to be an important regulator of synaptic glutamate receptor mobility. Indeed, we find that the disruption of spine maturation due to decreased CYFIP1 levels also correlates with an increase in AMPA receptor mobility within the spine postsynaptic density. Consequently, CYFIP1 expression levels appear to be critical not just for the maturation of dendritic spines but may also be important for regulating the subsynaptic cytoskeletal architecture essential for the stabilization of postsynaptic AMPA receptors.

Dynamic regulation of F-actin assembly is repeatedly implicated in mechanisms underlying spine structural plasticity.^21,56, 57, 58, 59, 60, 61^ We observe that NMDA receptor-induced spine remodelling no longer occurs in *Cyfip1*^+/^^−^ neurons, suggesting that when CYFIP1 levels are reduced, F-actin assembly is basally overactive and cannot induce further spine remodelling in response to NMDA. CYFIP1 has been previously shown to couple local actin polymerization to membrane budding at subcellular compartments such as the trans-Golgi network.^33,62^ Therefore, it will be interesting in the future to determine if the coupling of activity-dependent AMPA receptor trafficking (e.g., at the Golgi or in the endocytic network) to alterations in spine volume is also disrupted upon CYFIP1 depletion.^63^

Our results suggest that CYFIP1 is part of a growing number of mental illness-associated actin-regulatory molecules, including DISC1, DTNBP1, SHANK3, SRGAP2, Oligophrenin and the Abi1-WAVE2 complex that act in Rac1-dependent pathways to regulate dendritic development and spine dynamics.^24, 25, 26, 27, 28, 29^ As we observe an increase in immature spines upon CYFIP1 overexpression or haploinsufficiency *in vitro*, it is interesting to note that several other proteins known to signal via actin-regulatory pathways have been shown to drive similar effects on spine structure when either up- or downregulated, including VCP/neurofibromin, NESH/Abi-3 and Cofilin.^47,48,57,64, 65, 66, 67^ Thus, the precise tuning of CYFIP1 expression levels may be crucial for stabilising synaptic contacts, and skewing its levels above or below a critical threshold may force spines into a structurally unstable state. This may explain why both deletions and duplications of CYFIP1 have been linked to major mental illness.

Recent work has highlighted that rare complete gene knockouts in humans have a significant role in ASD and major mental illness.^68^ Although both deletions and duplications of CYFIP1 have been identified, total loss of CYFIP1 in humans seems unlikely as we report here that complete CYFIP1 deletion in mice leads to a failure in early embryonic development with *Cyfip1*-null embryos unable to survive beyond E8.5. Similar developmental abnormalities, resulting in death during early embryogenesis, have been observed in mice lacking other critical actin-regulatory genes such as *Rac1* and the WAVE complex component *Nap1*. Deletion of these genes in the mouse cause death during gastrulation at E7.5 and E9, respectively.^69, 70, 71^ Therefore, throughout early mouse development, CYFIP1 signalling events, probably via its core conserved actin-regulatory functions, are likely critical for gastrulation and normal patterning of embryonic structure in addition to later roles in neuronal development and plasticity.^72^ The embryonic lethality observed in the CYFIP1 knockout suggests that CYFIP2 cannot compensate for all CYFIP1 functions. Intriguingly, we also observed CYFIP2 to be enriched in dendritic spines and its overexpression could drive increased dendritogenesis. This suggests that it will be interesting to further explore whether CYFIP2 is a candidate susceptibility gene for major mental illness.

Our results provide important new insights into the role CYFIP1 has in the development of the nervous system and how *Cyfip1* dysregulation can alter CNS function to contribute to the development of neuropsychiatric illness. Changes in the structure and function of excitatory synapses, and alterations in the local versus long-range dendritic connectivity of neurons, have both been associated with the development of neuropsychiatric conditions involving imbalances of excitation and inhibition, such as ASD and SCZ.^73,74^ The roles for CYFIP1 described here may help inform how altered levels of this molecule gives rise to network-level dysfunction that define these pathologies.
