# Supplementary figures and images for: The autism and schizophrenia associated gene CYFIP1 is critical for the maintenance of dendritic complexity and the stabilization of mature spines
Source: Transl Psychiatry. 2014 Mar 25;4(3):e374–. doi: 10.1038/tp.2014.16 (PMC3966042; doi:10.1038/tp.2014.16)

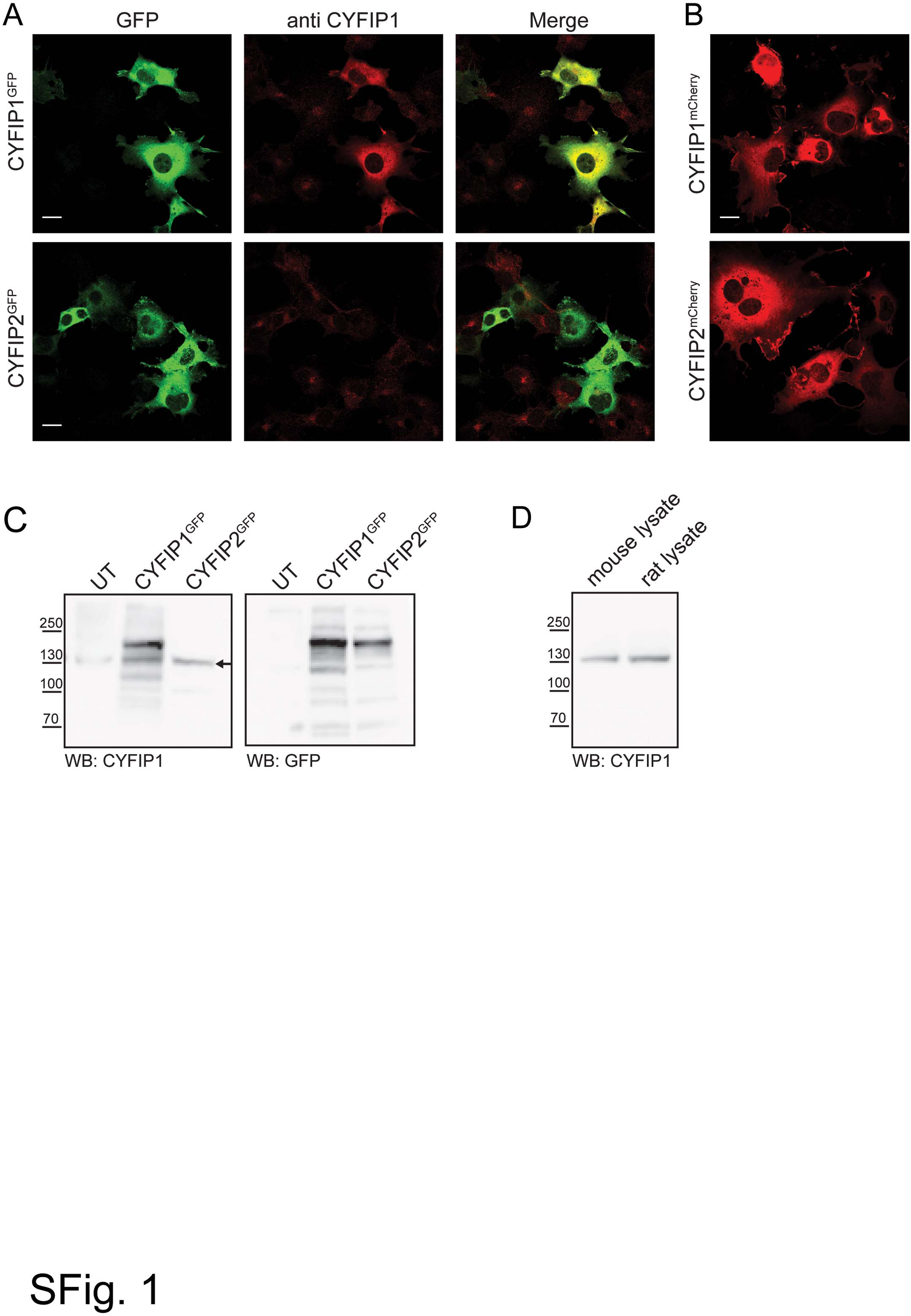

Supplement: Supplementary Figure 1 [file tp201416x1.tif]

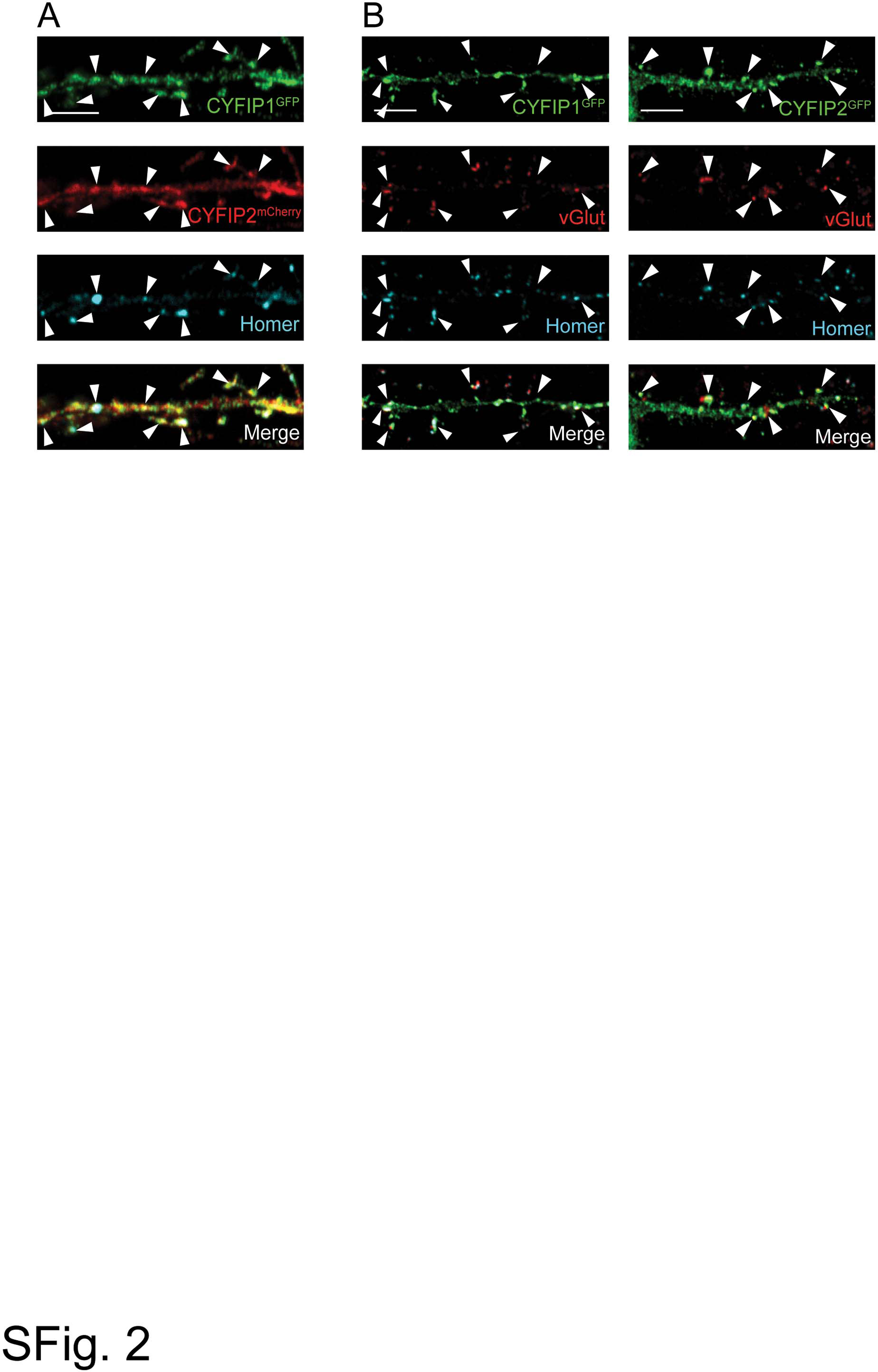

Supplement: Supplementary Figure 2 [file tp201416x2.tif]

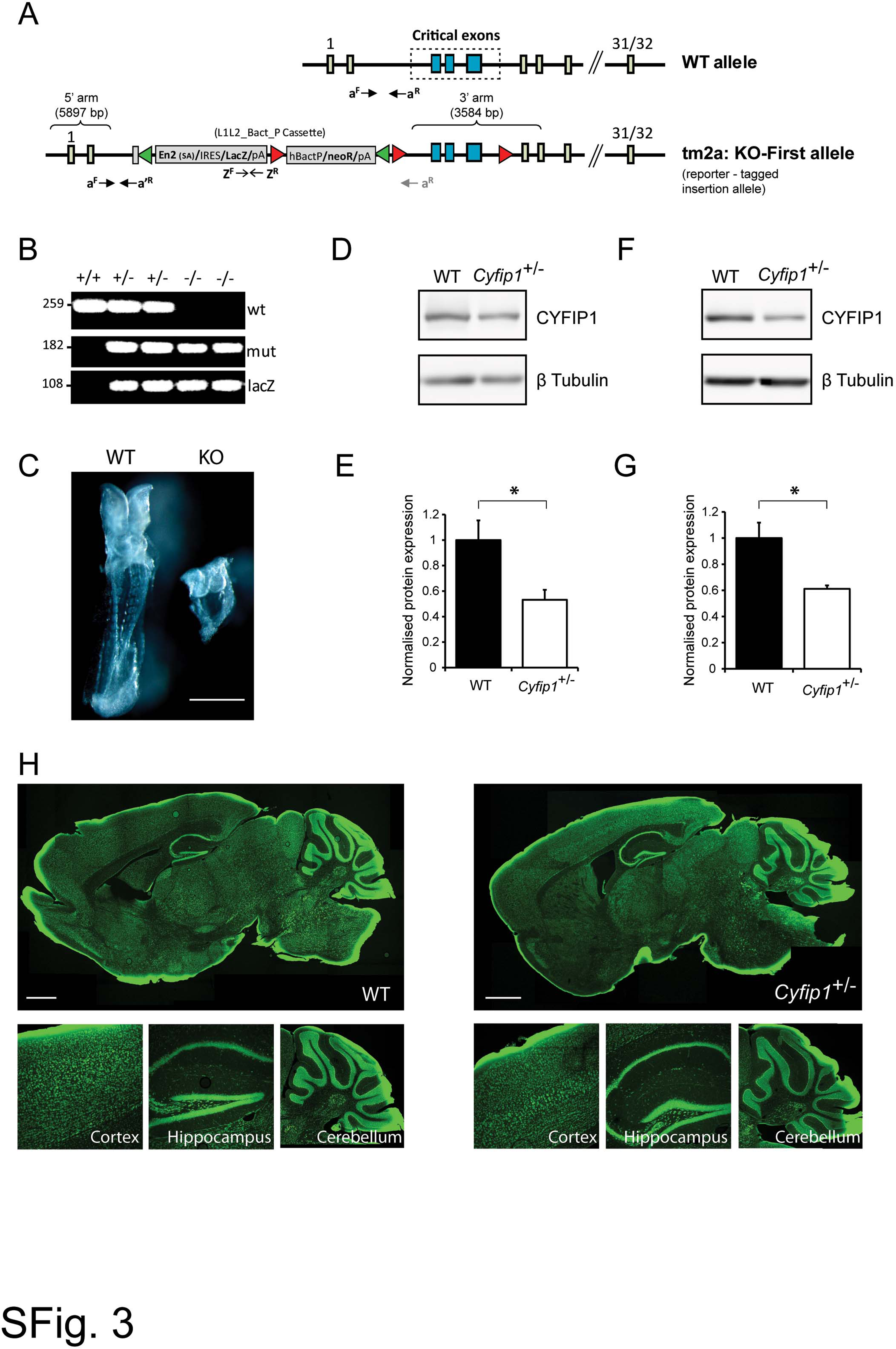

Supplement: Supplementary Figure 3 [file tp201416x3.tif]

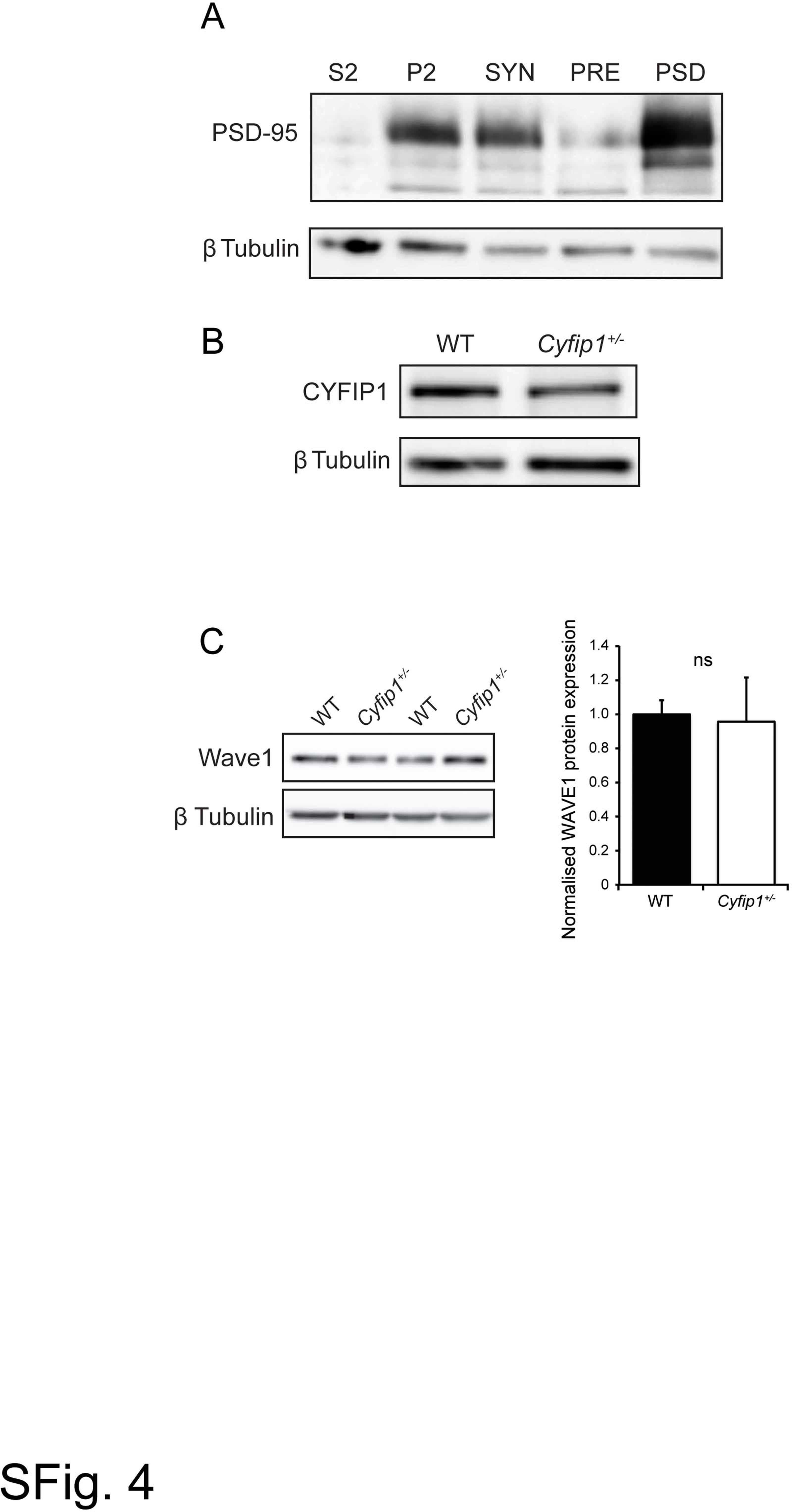

Supplement: Supplementary Figure 4 [file tp201416x4.tif]

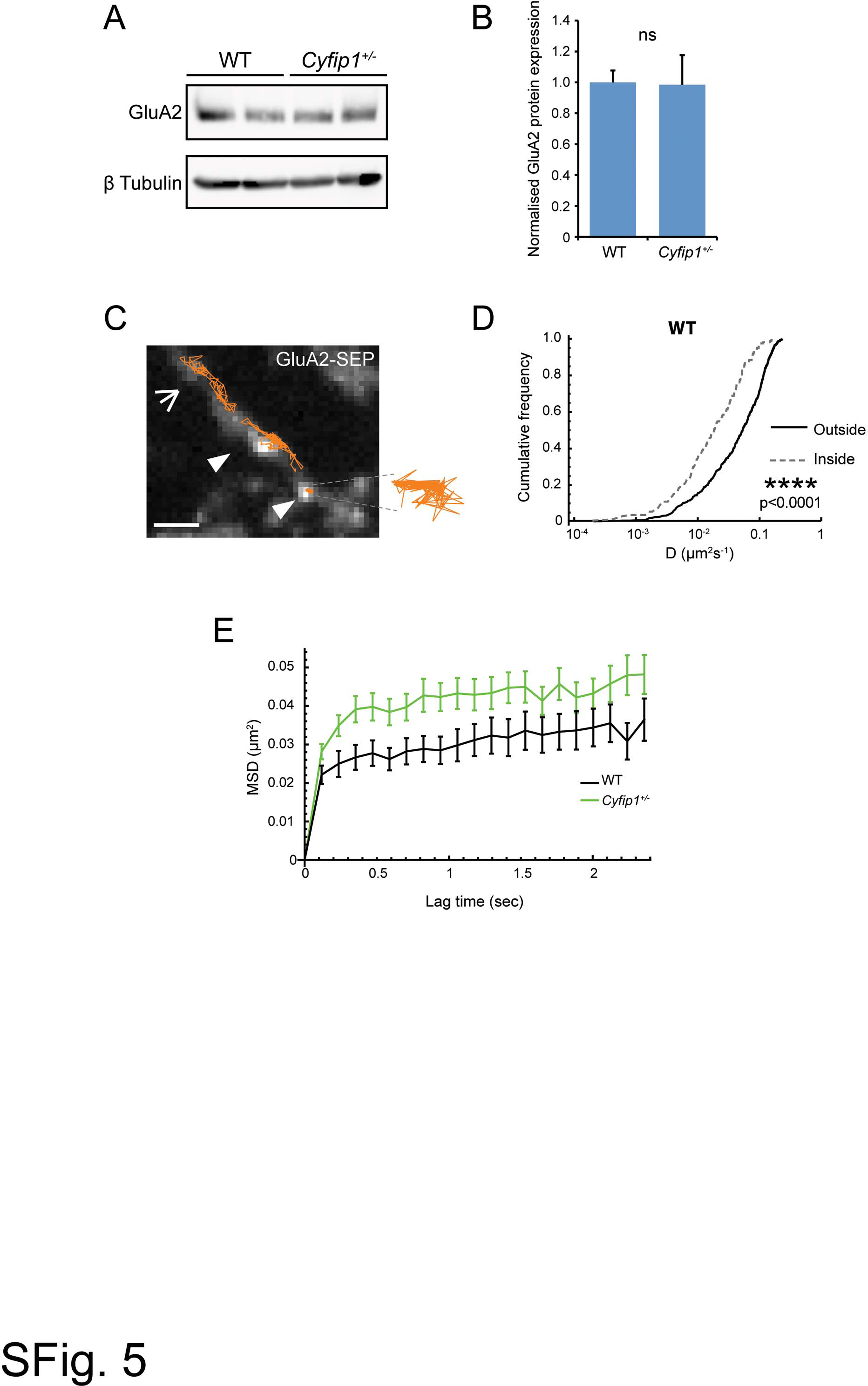

Supplement: Supplementary Figure 5 [file tp201416x5.tif]

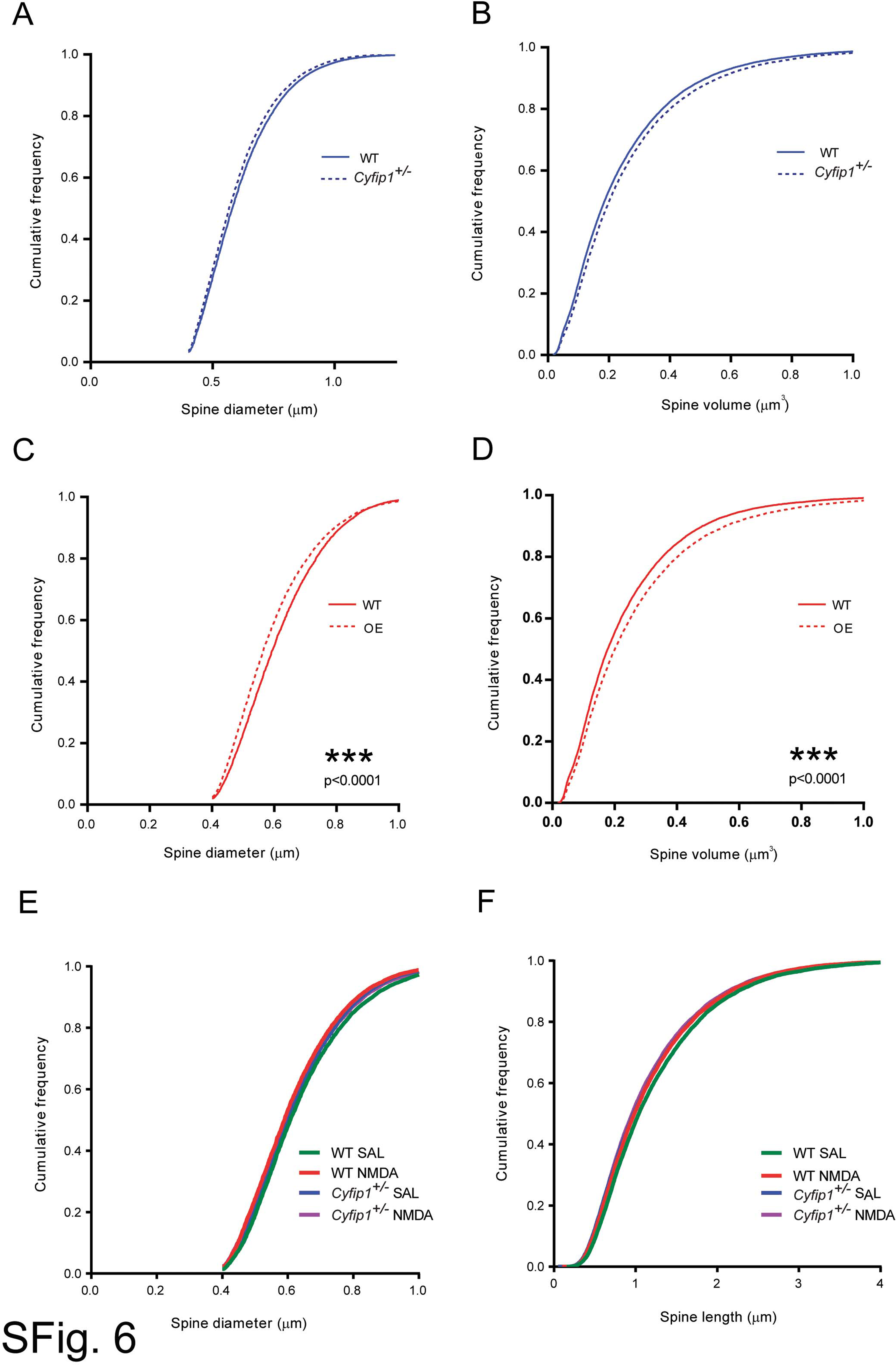

Supplement: Supplementary Figure 6 [file tp201416x6.tif]

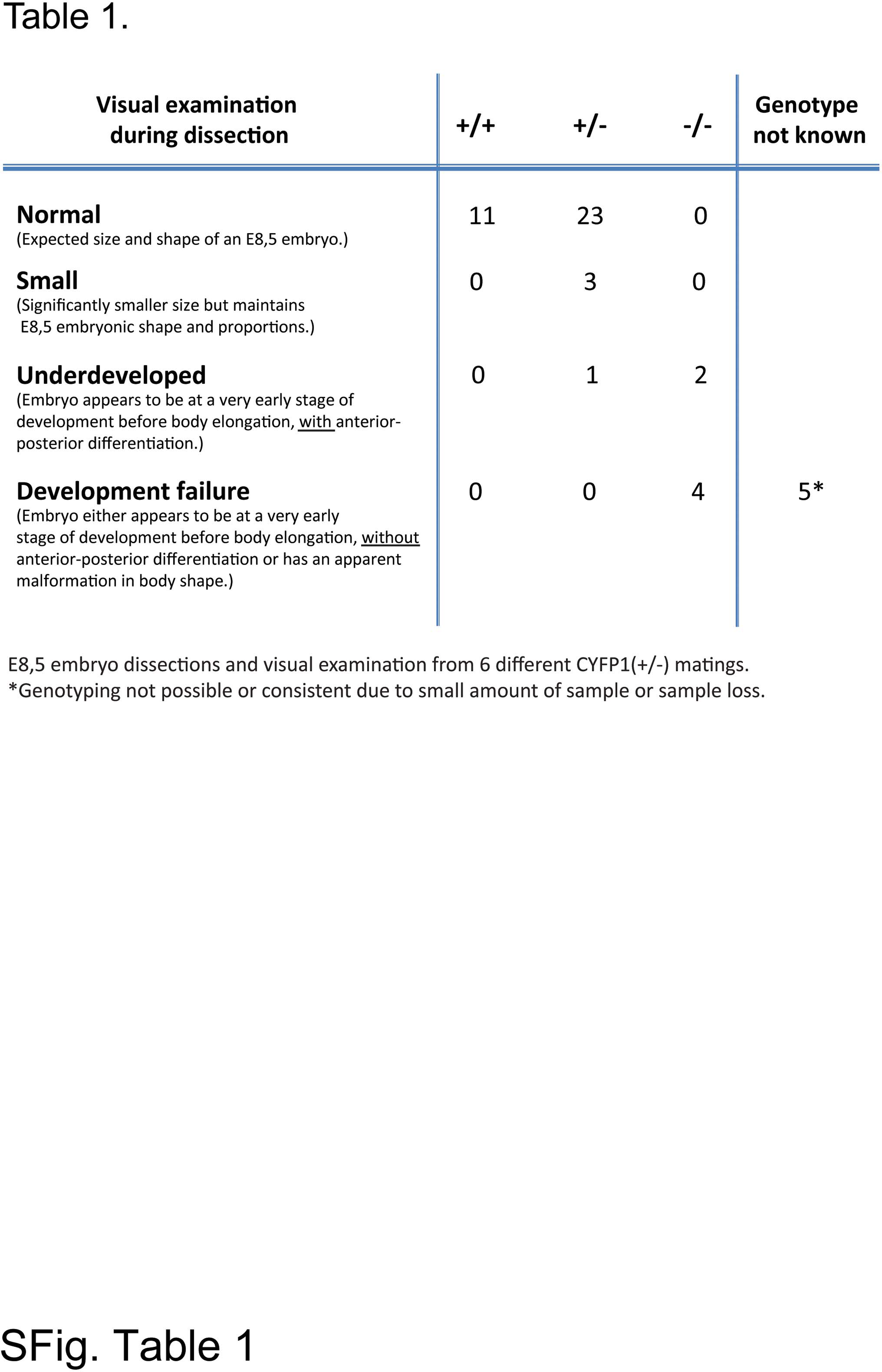

Supplement: Supplementary Table 1 [file tp201416x8.tif]
