# Supplementary material for: The autism and schizophrenia associated gene CYFIP1 is critical for the maintenance of dendritic complexity and the stabilization of mature spines
Source: Transl Psychiatry. 2014 Mar 25;4(3):e374–. doi: 10.1038/tp.2014.16 (PMC3966042; doi:10.1038/tp.2014.16)
Supplement: Supplementary Figure Legends [file tp201416x7.doc]

**SFigure1. Confirmation of CYFIP1 and CYFIP2 fusion protein expression and characterisation of a CYFIP1 specific antibody.**

Transfection of COS-7 cells with human CYFIP1 or CYFIP2 **(A)** GFP or **(B)** mCherry-tagged constructs. Strong GFP and mCherry signal confirms that these fusion proteins are readily expressed. Immunostaining of CYFIP1^GFP^ or CYFIP2^GFP^ transfected cells with a commercial anti-CYFIP1 antibody (Upstate) shows the antibody is specific to CYFIP1 alone **(A)**. Scale bar, 20 μm. **(C)** Western blotting of untransfected (UT), CYFIP1^GFP^ or CYFIP2^GFP^ COS-7 cell lysates and probing for GFP (right panel) confirms that these constructs generate fusion proteins of the expected molecular weight ~175 kDa. Probing for CYFIP1 (left panel) further confirms the specificity of the anti-CYFIP1 antibody producing a specific band for CYFIP1^GFP^ alone at ~175 kDa and a non-specific band at ~130 kDa present in all the lanes (arrow). **(D)** Probing mouse and rat brain lysate on a western blot with the CYFIP1 specific antibody confirms the antibody reacts to both rat and mouse endogenous CYFIP1 producing a band at the expected molecular weight ~145 kDa.

**SFigure2. CYFIP1 and CYFIP2 localise together in dendrites and localise to excitatory postsynaptic sites.**

Rat hippocampal neurons were transfected at 17DIV with CYFIP1 and/or CYFIP2 constructs and immunostained at 20DIV. **(A)** Neurons co-expressing CYFIP1^GFP^ and CYFIP2^mCherry^ and stained for the excitatory postsynaptic marker Homer confirm that CYFIP1 and CYFIP2 occupy the same clusters in dendrites and spines and co-localise with excitatory postsynaptic sites (arrowheads). When individually expressed in neurons CYFIP1^GFP^ and CYFIP2^GFP^ colocalise with the excitatory postsynaptic marker Homer opposed to the presynaptic marker VGLUT (arrowheads) **(B)**. Scale bars, 5μm.

**SFigure3. Generation of *Cyfip1* knockout and *Cyfip1* happloinsufficient mice.**

**(A)** Design of the knockout (KO)-first allele system showing the wild-type (WT) allele; the KO-first *Cyfip1* allele (tm2a allele) containing an IRES:*lacZ* trapping cassette and a floxed promoter-driven *neo* cassette inserted between exons 2 and 3 of *Cyfip1*, disrupting gene function. Both cassettes are bound by two frt sites (green triangles). The neo cassette and 3’ frt site are flanked by loxP sites with an additional distal loxP site present 3’ of exon 5 (red triangles). *Cyfip1* KO animals had two KO-first alleles while happloinsufficient animals were heterozygous for the KO-first allele (41,75). **(B)** PCR analysis of F1 progeny from parental mice heterozygous for the KO-first allele. Animals were genotyped with the primers a^F^ and a^R^ to produce a PCR product of 259 base pairs (bp) from the WT allele, these primers were too distant from each other to produce a product from the KO-first allele. Primers a^F^ and a^’R^ produced a 182 bp product from the KO-first allele with a^’R^ annealing at the very 5’ region of the *lacZ* cassette. Primers Z^F^ and Z^R^ produced a 108 bp product from the KO-first allele within the introduced *lacZ* gene. Lane 1 is a WT mouse (+/+) positive for just the WT product, lanes 2 and 3 are heterozygous animals (+/-) positive for both the WT allele and the KO-first allele PCR products, lanes 4 and 5 are KO animals with no WT PCR product present (-/-). **(C)** Representative images of *Cyfip1* WT and KO embryos at E8.5 highlighting the developmental defects seen in KO animals. Scale bar, 0.5mm. Western blot analysis and quantification displaying fold change of CYFIP1 protein levels from control (WT) and *Cyfip1^+/-^* P55 hippocampal brain lysates **(D,E)** or DIV16 cultured cortical lysates **(F,G)**. Both hippocampal brain lysates and cortical neurons from *Cyfip1*^+/-^ animals had ~40% less CYFIP1 protein compared to WT controls (Brain lysate: 46.8% CYFIP1 protein decrease, WT control 100% ± 15.4%, *Cyfip1*^+/-^ 53.2 ± 7.8%, n=3-4, *p<0.05; cortical neurons: 38.9% CYFIP1 protein decrease, WT control 100% ± 11.8%, *Cyfip1*^+/-^ 61.1% ± 2.6%, n=3-4, *p<0.05). **(H)** Nissl staining of WT and *Cyfip1^+/-^* adult mouse sagital brain sections shows no major change in gross brain morphology between the two genotypes.

**SFigure 4. PSD fractionation demonstrates CYFIP1 is present at excitatory synapses.**

WT and *Cyfip1*^+/-^ PSDs were prepared as described in *Supplementary Methods* from animals aged P40-60. **(A)** shows PSD-95 accumulation in the PSD fraction, indicating successful enrichment of PSDs using this protocol. **(B)** shows that CYFIP1 is present in PSD fractions from WT neurons and is reduced in PSD fractions from *Cyfip1*^+/-^ neurons. Identifying biochemically the presence of CYFIP1 in the PSD agrees with our immunocytochemical data showing CYFIP1 enrichment at excitatory synapses. **(C)** Western blot analysis and quantification of WAVE1 total protein levels from control (WT) and *Cyfip1^+/-^* DIV16 cultured cortical lysates (n=3).

**SFigure 5. GluA2 receptor diffusion dynamics inside and outside synaptic clusters**

**(A)** Western blot analysis and **(B)** quantification of the AMPA receptor subunit GluA2 total protein levels from control (WT) and *Cyfip1^+/-^* DIV14-16 cultured cortical lysates. Neurons showed no difference in protein levels (n=4). GluA2^SEP^-containing receptors in WT neurons were labelled with quantum dots (QDs) and live imaged, representative QD trajectories are shown in **(C),** synaptic trajectories (filled arrowheads), extrasynaptic trajectory (open arrowhead). Scale bar, 2μm. **(D)** QD-labelled, GluA2^SEP^-containing AMPA receptors are slower inside synaptic clusters than outside in WT neurons, indicating that their mobility is restricted at synapses (n_in_=164 n_out_=514 QD tracks, ****p<0.0001). **(E)** Mean squared displacement (MSD) analysis of GluA2^SEP^ containing receptor trajectories inside synapses in WT and *Cyfip1^+/-^* neurons. The sublinear curve is characteristic of synaptic confinement; receptors are less confined in *Cyfip1^+/-^* neurons as indicated by the upward shift of the *Cyfip1^+/-^* curve.

**SFigure 6. Spine metrics following *Cyfip1* haploinsufficiency, *Cyfip1* overexpression and following chemical LTD in *Cyfip1* haploinsufficient neurons**

Spine morphology was analysed at 21DIV in *Cyfip1*^+/-^ neurons and compared to WT cells **(A,B).** *Cyfip1*^+/-^ neurons showed no significant difference in spine diameter or volume. Spine morphology was analysed at 21DIV in CYFIP1^mCherry^ overexpressing neurons and compared to cells expressing DsRed alone **(C,D).** CYFIP1^mCherry^ overexpressing neurons showed a small but significant decrease in spine diameter and a significant increase in spine volume, (n=15000-19000 spines per condition, ***p<0.001). Spine diameter and length are unchanged following NMDA treatment in both WT and *Cyfip1*^+/-^ neurons **(E,F)**.
